# Vitamin D and Atopic Dermatitis—A Mere Correlation or a Real Supportive Treatment Option?

**DOI:** 10.3390/nu17162582

**Published:** 2025-08-08

**Authors:** Kamil Przechowski, Marta Natalia Krawczyk, Rafał Krasowski, Rafał Pawliczak, Paulina Kleniewska

**Affiliations:** Department of Immunopathology, Faculty of Medicine, Medical University of Lodz, Żeligowskiego 7/9, 90-752 Łódź, Poland

**Keywords:** vitamin D, atopic dermatitis, observational studies, animal studies, randomized controlled trials, vitamin D deficiency, 25-hydroxyvitamin D, VDR polymorphism

## Abstract

Atopic dermatitis (AD), a common chronic inflammatory disease in children and adults, is often studied to find the best way to prevent or reduce its severity. One of the substances tested so far is vitamin D. The main aim of this paper was to determine whether vitamin D truly brings benefits to people with AD or whether its action is too insignificant to have clinical significance. The review covered articles—observational studies, several animal studies and randomized controlled trials (RCTs)—available in the PubMed database and published after 2019. Full-text manuscripts in English were used. Observational studies presented both therapeutic effects of vitamin D and its lack of influence on AD. They also determined that vitamin D receptor (VDR) polymorphism may indeed affect the occurrence and severity of this disease. Similarly, the results of vitamin D’s effect on AD are inconclusive in RCTs. Meanwhile, animal studies showed only the attenuation of disease symptoms in mice. The still-growing number of studies on vitamin D and its association with AD, due to many internal and external distorting factors, has not been able to provide us with definitive results. It is necessary to conduct further appropriately designed large-scale studies, including long-term observation.

## 1. Introduction

Although the number of scientific publications on the relationship between blood vitamin D levels, vitamin D supplementation, and the development of atopic dermatitis (AD) is steadily increasing, the findings of existing studies remain inconclusive [1,2,3,4,5,6,7,8,9,10,11,12,13,14,15]. The results of recently published randomized controlled trials (RCTs) assessing the effect of vitamin D supplementation on the course of AD show significant discrepancies [3,4,5,6,7,8]. Also, the latest systematic reviews and meta-analyses indicate both a possible therapeutic effect of supplementation and a lack of statistically significant differences compared to placebo groups [9,10,11,12,13,14,15]. AD is one of the most common chronic skin diseases in children, its incidence is also reported in a significant number of adult patients, but there are significant differences in the course of the disease in the two age groups [16,17,18,19]. The disease is characterized by its chronic and relapsing nature, and the most bothersome symptoms include intense itching and dryness of the skin, which markedly reduce the comfort of patients’ lives [16,17,18]. The mechanisms responsible for the development of AD are multifactorial and include both deficits in epidermal barrier function and the predominant activity of the Th2-type immune response [20,21,22,23]. Th2 cytokines play a key role in the pathogenesis of AD, particularly IL-13, whose expression in the skin dominates IL-4, while the importance of IL-5 remains ambiguous, despite its association with eosinophilia and disease activity [20]. Vitamin D, due to its immunomodulatory properties, may beneficially influence the course of AD by inhibiting the activity of immune cells (macrophages and T lymphocytes) that are involved in the development of the inflammatory response [24,25]. Furthermore, inadequate vitamin D levels have been increasingly associated with an increased incidence of AD [26,27,28].

This article aims to synthesize the current state of research on the role of vitamin D supplementation in the context of AD treatment. The paper is based on a search of the PubMed database for publications published since 2019, with a focus on RCTs, as well as observational studies, animal studies, systematic reviews, meta-analyses, and traditional reviews. Full-text manuscripts in English were used.

## 2. Vitamin D

Vitamin D has become a key public health issue, as its deficiency affects people of all ages and sexes [29,30]. It is caused both by limitations in sunlight and insufficient dietary intake [31,32]. Particular risk groups are dark-skinned individuals, pregnant women, the elderly and obese, and patients with chronic kidney disease [33,34,35,36,37]. Values below 10–12 ng/mL are considered severe vitamin D deficiency, while levels above 30 ng/mL are seen as sufficient or normal [38,39]. Current scientific data suggest that the addition of vitamin D to commonly consumed foods, such as dairy products, orange juice or bread, can significantly increase its serum levels [40,41,42]. On the other hand, Lehtonen-Veromaa et al. [43] did not detail the enrichment of liquid dairy drinks and margarines with vitamin D for the prevention of vitamin D deficiency.

### 2.1. Molecular Characterization of Vitamin D

Vitamin D compounds are a large family of cholesterol derivatives that belong to the steroid hormones and undergo numerous enzymatic and non-enzymatic transformations [44,45]. They have a secosteroid structure, meaning that one of their ring structures has been cleaved by a bond break [45]. The two most relevant forms are ergocalciferol (D2) and cholecalciferol (D3), which differ in their side chain structure and site of synthesis [45]. Vitamin D3 is synthesized in the skin under UVB from 7-dehydrocholesterol by previtamin D3, which then isomerizes, whereas D2 is synthesized in yeasts and other fungi [44,45,46].

### 2.2. Vitamin D Metabolism

**Figure 1:**
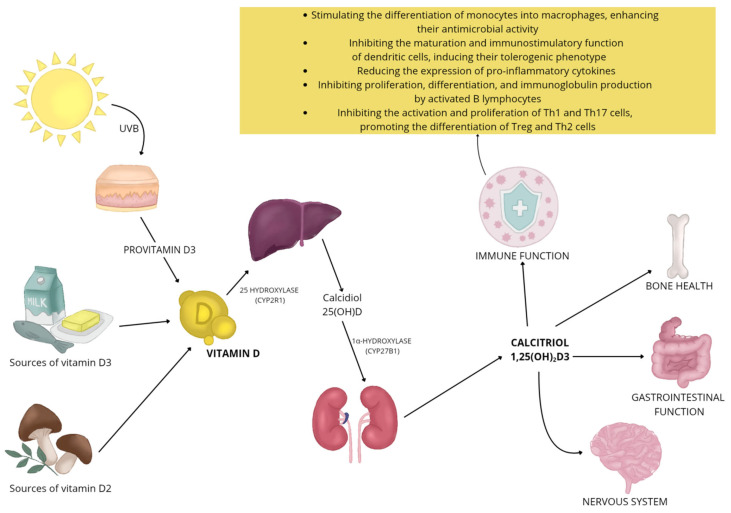
Vitamin D functions and metabolism.

Once in the body, vitamin D undergoes a two-step activation (hydroxylation): first in the liver, where it is converted to the 25(OH)D form (calcidiol), and then in the kidneys, where the attachment of another hydroxyl group leads to the formation of active 1,25(OH)2D3 (calcitriol) (Figure 1) [45,46]. Various enzymes of the cytochrome P450 family are involved in this process [45,46,47]. Vitamin D metabolites are carried in the bloodstream mainly in association with vitamin D-binding protein (VDBP) and albumin, with only a small proportion occurring in unbound, or free, form [46,48]. Vitamin D inactivation occurs primarily with the enzyme 24-hydroxylase (CYP24A1) [45]. It catalyzes the metabolism of both 25-OH-D3 and its active form, i.e., 1,25(OH)2D3, leading to the formation of a number of metabolites hydroxylated at positions 24 and 23, and consequently leads to the formation of biologically inactive biliary compounds, including calcitroic acid [45,47].

### 2.3. Effects of Vitamin D

The action of vitamin D extends far beyond its classical functions related to bone mineralization and maintenance of calcium-phosphate homeostasis [49,50]. It plays an important role in the regulation of many other body systems, including the nervous system [51], the function of skeletal muscles [52], the maintenance of gastrointestinal homeostasis [53], and regulates the immune system (Figure 1) [54]. The active form of vitamin D interacts with cells primarily through the VDR, which, as a nuclear transcription factor, regulates gene activity in a cell-type-differentiated manner [55]. In addition, vitamin D can rapidly induce cellular responses unrelated to gene expression by engaging membrane receptors, indicating its broad and multidirectional biological effects [55]. Deficiencies of this vitamin are associated with an increased risk of many conditions [56], such as cardiovascular diseases [57], neurodegenerative diseases [58], or cancer [59].

## 3. AD as a Health and Social Problem

AD is a chronic, relapsing inflammatory skin disease, mainly affecting children [21]. Patients have to deal with numerous bothersome symptoms on a daily basis, such as dry and itchy skin, impacting their quality of life significantly by affecting their mental health, interpersonal relationships or decisions related to career and family life [16,17]. A chronic course of the disease accompanied by constant itching and scratching can lead to thickening and lichenification of the skin [21]. Findings indicate significant associations between AD and other conditions [60]. Ravnborg et al. [61] found that the prevalence of asthma among people with AD was as high as 25.7% (95% CI, 23.7–27.7), more than three times the risk compared to the general population (odds ratio, 3.03; 95% CI, 2.64–3.47). A cohort study showed that 61.7% of European patients additionally have sleep problems and difficulties, and up to 75.9% suffer from depression [62].

It is estimated that globally, about 20% of children and up to 10% of adults suffer from AD [18]. A variety of environmental and genetic factors influence the prevalence of AD [63]. Findings have detailed, among others, the effects of air pollution [64,65,66] or tobacco smoke [67] increase the risk of AD. Exposure to particulate matter of 2.5 μm or less (PM2.5) in the home environment has been observed to exacerbate AD symptoms in pediatric patients, especially in those with current inhalant allergies and a severe disease course [66]. Shin et al. [68] suggest that urban residence is associated with a significant increase in the risk of AD, especially in children, in whom the chance of developing the disease was approximately 1.5 times higher than in children living outside the city (95% CI, 0.99–1.67).

### 3.1. Pathogenesis of the Disease

**Figure 2:**
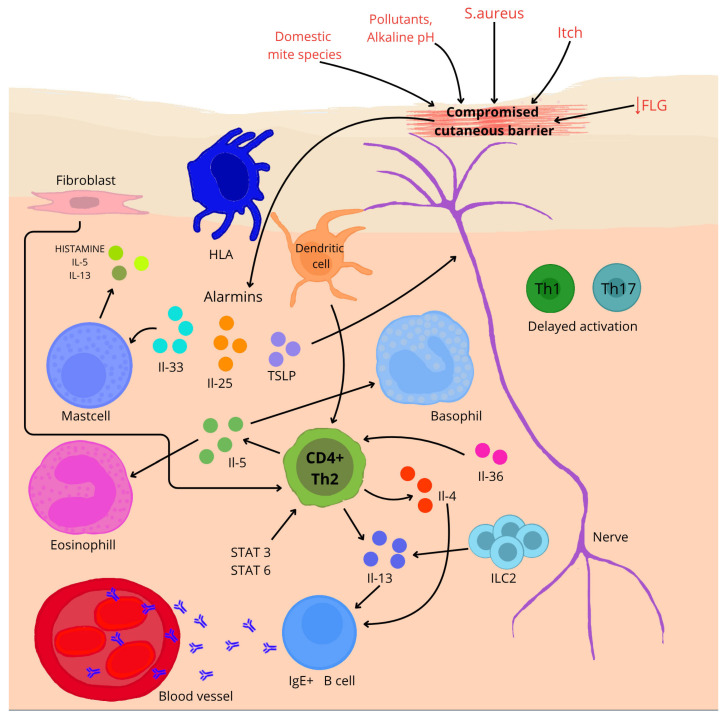
A brief overview of the pathogenesis of AD. ↓—reduction; arrows—influence on (depending on direction).

AD is a chronic skin disease with heterogeneous pathogenesis, involving both epidermal barrier dysfunction and immune dysregulation, caused by environmental and genetic factors (Figure 2) [20,21,22,23]. The integrity of the skin barrier plays a key role in the pathogenesis of AD. The protein responsible for maintaining this integrity is filaggrin (FLG). Defects in this protein result in dry skin (reduction in the natural moisturizing factor), itching, hypersensitivity, susceptibility to irritants or allergens, and an increased risk of infection. As well as human and animal model studies confirm that loss-of-function variants of the FLG gene lead to an impaired skin barrier, which increases susceptibility to the development and more severe course of AD [69,70,71,72]. Hoyer et al. [71] observed that infants carrying mutations in the gene encoding the FLG protein have a more than 3.5 times increased risk of developing AD by 1 year of age (OR 3.66, 95% CI 2.44–5.48, *p* < 0.001). The results are also supported by an evaluation by Srinivas et al. [72], who identified 16 novel FLG loss-of-function variants, and their presence significantly correlated with early disease onset among Indian children before 1 year of age (rho = −0.277; 95% CI: −0.475 to −0.0539; *p* = 0.016); up to 65.4% of carriers of these variants developed the disease in infancy. Other studies [73,74,75] detail decreased expression of claudin-1 (CLDN1), an elementary factor of tight epidermal junctions, as a component contributing to increased skin permeability and enhanced inflammatory response. Additionally, according to transcriptomic analysis conducted by Zhou et al. [76], Th2 cytokines may lead to reduced expression of IL-37. In turn, IL-37 protein has been shown to increase the expression of FLG and FLG2 proteins (*p* = 0.056; *p* < 0.05) in the outer layer of the epidermis.

In AD, epidermal barrier function is impaired, allowing allergens and pathogens to penetrate. Impaired keratinocytes release alarm cytokines such as IL-25, IL-33 and thymic lymphopoietin (TSLP), which initiate a Th2-type immune response [21,77]. ILC2 lymphoid cells (ILC2) are the key effectors of the Th2-type response in AD. Studies have shown that ILC2 numbers are increased in the skin of AD patients as well as mouse models. Their activity correlates with the severity of disease symptoms [78]. In a study on mouse models exposed to ovalbumin [79], it was confirmed that IL-25 produced by keratinocytes in response to damage activated ILC2, which subsequently produced IL-13 (*p* < 0.005), enhancing inflammation characteristic of AD. Single-cell analysis conducted by Alkon et al. [80] showed that ILC2 in the skin of patients with AD is capable of immunological plasticity. These cells, in addition to producing cytokines typical of the Th2 response, such as IL-4 and IL-13, can also produce cytokines specific to ILC3, for example, IL-17A, IL-22, and IL-26. This highlights the complexity of the immune system during the course of the disease.

Pro-allergic cytokines IL-4 and IL-13 play a central role in the pathogenesis of AD [21,77]. Both cytokines strongly suppress the expression of skin barrier proteins—filaggrin, loricrin, and involucrin—through activation of the STAT6/STAT3 pathways, which is directly associated with impaired epidermal integrity [81]. Moreover, studies using transgenic models have demonstrated that IL-4 not only amplifies the inflammatory response but also plays a crucial role in initiating the AD phenotype—leading to the development of characteristic skin lesions, such as pruritus, infiltration of immune cells, and elevated IgE levels—whereas its absence correlates with improved barrier function and increased expression of genes from the epidermal differentiation complex (EDC) [82,83].

In the chronic phase of AD, a mixed immune response is observed, involving Th1, Th17, and Th22 lymphocytes, whose cytokines—such as IFN-γ, IL-17, and IL-22—contribute to the persistence of inflammation, deterioration of skin barrier function, and exacerbation of clinical symptoms [84]. The importance of IL-17 in these processes is confirmed by studies in IL-17 knockout mice, in which the oxazolone-induced AD model showed significantly reduced ear swelling, decreased inflammatory infiltration in the skin, and lower levels of Th2 cytokines, as well as improvements in barrier function parameters, such as transepidermal water loss and lipid distribution in the stratum corneum [85]. In the chronic form of AD, IL-22 simultaneously suppresses the expression of inflammatory genes upon neutralization and accelerates wound healing in vitro. However, its interaction with TNF-α markedly amplifies the induction of pro-inflammatory genes—by as much as 5–15-fold—indicating its key role in both tissue regeneration and the maintenance of skin inflammation [86].

### 3.2. AD and Food Allergies

Atopic diseases often follow a characteristic progression known as the atopic march, which typically begins with AD in early childhood and may subsequently lead to other allergic conditions, such as food allergy (FA), allergic rhinitis, or bronchial asthma [87,88]. Both AD and FA are part of the same group of atopic disorders and share overlapping pathophysiological mechanisms, including immune system dysregulation and alterations in the microbiome. These simultaneous processes may mutually exacerbate each other [89,90,91].

The association between AD and FA has been well documented. It is estimated that approximately one-third of patients with AD suffer from food allergies (95% CI: 28.8–36.6), while AD symptoms occur in nearly half of individuals with confirmed FA (95% CI: 41.4–49.3) [92], indicating a clear bidirectional relationship. A systematic review of 66 studies further supported this connection, revealing that in population-based studies, infants with AD at the age of 3 months had up to a six-fold increased risk of developing FA (OR: 6.18; 95% CI: 2.94–12.98; *p* < 0.001), and the prevalence of food allergy confirmed by challenge tests reached as high as 81% in AD patients [93].

Epidemiological data show that early onset of AD (before age 2) significantly increases the likelihood of developing FA, and persistence of AD symptoms until the age of 6 can elevate this risk up to 7.8 times compared to children without AD (95% CI: 3.42–17.73) [94]. Moreover, children with AD who are sensitized to more than 10 food allergens tend to experience a more severe disease course, as indicated by higher transepidermal water loss (TEWL: 27.6 ± 8.7 g/m^2^/h), reduced skin hydration, and elevated the scoring atopic dermatitis (SCORAD) index (*p* < 0.005) [95].

The most frequently identified allergens in children with AD are hen’s eggs (52.63%; *p* = 0.1887) and dairy products (43.86%; *p* = 0.1204) [96]. Additionally, infants with atopic eczema are at a significantly increased risk of sensitization to peanuts—up to eleven times higher than in children without skin lesions (95% CI: 6.6–18.6) [97].

## 4. The Link Between Vitamin D and AD

Vitamin D, through various molecular pathways, likely exerts significant effects on skin cells and the immune system in AD, modulating both the integrity of the epidermal barrier and the inflammatory response [1]. The authors [98] showed that administration of calcifediol to mice with induced AD significantly reduced keratinocyte proliferation and inflammatory cell infiltration, which was associated with inhibition of STAT3 phosphorylation and the AKT/mTOR signaling pathway, as well as with a reduction in the expression of the aquaporin 3 (AQP3) protein channel, which is crucial for maintaining the water balance of the epidermis. Similar effects were observed in a study [99] using calcitriol in NC/Nga mice, where researchers observed improved skin barrier integrity and reduced inflammatory markers such as IL-13 and IL-33. Interestingly, in another study comparing topical vitamin D3 cream with betamethasone in ovalbumin-sensitized mice, vitamin D3 effectively lowered filaggrin levels, IgE antibodies, and IL-5 levels, while also significantly reducing epidermal thickening [100]. Furthermore, Hermann et al. [101] suggested that vitamin D3 reduces the expression of the FcεRI receptor on dendritic cells by blocking the binding of the transcription factor to the promoter region of its gene, thereby attenuating IgE-dependent signaling.

### Vitamin D Deficiency and AD Symptoms

A meta-analysis conducted by Fu et al. [12] showed that serum calcidiol levels in AD patients are significantly lower than in healthy individuals. Additionally, an evaluation of 14 studies including 1450 AD patients and 1009 healthy controls showed a cumulative mean difference (MD) of −8.18 (95% CI: −13.15, −3.22), and a cumulative effect size of Z = 3.23 (*p* = 0.001). SCORAD scores from 9 studies demonstrated that serum 25(OH)D concentrations in patients with severe AD were significantly lower than in patients with mild disease. In this case, the cumulative MD was 9.23 (95% CI: 6.92, 11.55), and a Z-effect size of 7.82 (*p* < 0.001). SCORAD scores from five studies and eczema area and severity (EASI) scores from three RCTs showed that after vitamin D supplementation, their values significantly decreased by approximately 11.2 and 3.72 points, respectively. Similar conclusions were drawn from a case–control study [102] in which 96 patients with atopic disease and 90 healthy controls without chronic diseases were diagnosed. In the patient group higher eosinophils and total IgE levels were observed, whereas vitamin D levels were markedly lower (*p* = 0.018; *p* = 0.001). In 56 patients a clear vitamin D deficiency was noted. A statistically significant (*p* = 0.001) moderate (r = 0.460) negative correlation between vitamin D and SCORAD was determined. Also, in non-atopic patients a statistically significant (*p* = 0.031) weak negative correlation (r = −0.305) between vitamin D and SCORAD was found. The presented results, as well as other available data [3,5,12,103,104,105], confirm the significant impact of vitamin D deficiency on AD severity. SCORAD and EASI values are generally elevated in individuals with this vitamin D deficiency.

**Figure 3:**
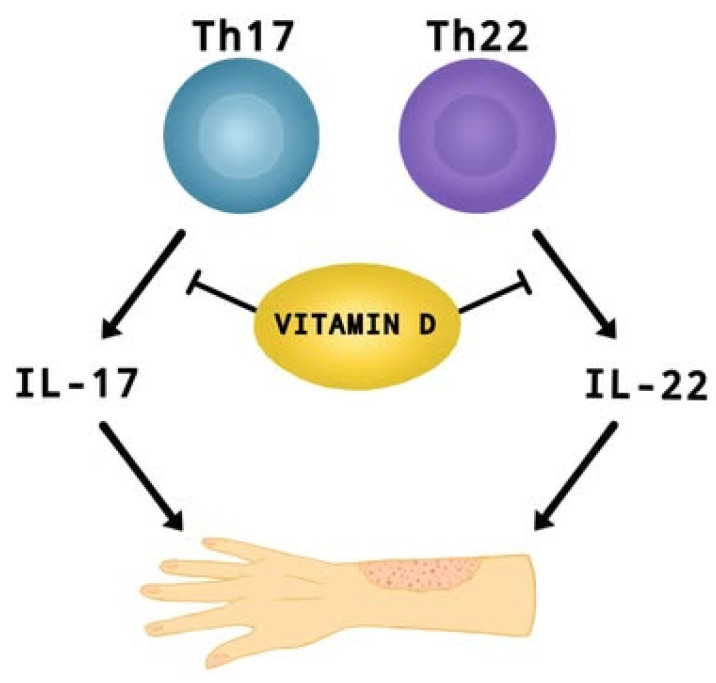
The effect of vitamin D on suppressing the production of IL-17 and IL-22 by Th17 and Th22 cells. These interleukins contribute to the development of AD.

In recent years, researchers have paid considerable attention to the role of vitamin D supplementation in alleviating the symptoms and impacting the severity of AD [3,5,8,9]. A meta-analysis conducted last year [9] including children and adults, with mild to moderate AD, showed that vitamin D supplementation leads to a significant reduction in AD severity compared to control groups (SMD = −0.41, 95% CI: −0.67 to −0.16, *I*^2^ = 58%, *p* < 0.01). Dogru [26] reports that pediatric AD patients (aged 5.6 ± 2.7) have lower mean 25OHD3 levels (19.86 ± 6.7 ng/mL) than healthy children—24.07 ± 9.08 ng/mL (aged 5.4 ± 23; *p* = 0.002). Identical findings were obtained for adults (aged 35.5 ± 13.7) [27], where the risk of AD was almost 1.5 times higher in those with lower than 12 ng/mL vitamin D levels (95% CI: 1.04–2.12; *p* = 0.02), as well as in infants aged 0–12 months [28], where up to 50% of patients were vitamin D deficient. Additionally, Barlianto et al. [28] reported a positive correlation between disease severity (SCORAD index), IL-17A (r = 0.522, *p* = 0.001) and IL-22 (r = 0.612, *p* < 0.001) levels, with an inverse relationship between SCORAD and vitamin D levels (r = −0.714; *p* < 0.001). This suggests that vitamin D may modulate the inflammatory response (Th17 and Th22) by affecting pro-inflammatory interleukins, which play an important role in the development of AD (Figure 3) [28,106,107]. Cross-sectional and clinical studies show that vitamin D deficiency correlates with higher symptom severity and increased asthma severity [9,26,27,28]. Ronceray et al. [108], in a study involving patients with AD (23 children (38%) with a mean age of 6.9 ± 4.8 yrs and 37 adults (62%) with a mean age of 35 ± 14 yrs), found that those with more severe disease had significantly lower 25(OH)D levels (15.9 ± 8.3 ng/mL) compared to those with mild to moderate AD (21.5 ± 8.2 ng/mL; *p* = 0.01). However, other studies indicate that there is no clear and significant correlation between vitamin D levels in children or adults and the severity of AD [109,110,111]. Importantly, studies [110,111] did not take into account key factor variables such as exposure to natural sunlight, age, gender, presence of the infection, vitamin D intake or use of AD treatments. Shafiq et al. [109] in a cross-sectional study conducted in Bangladesh found no significant relationship between disease severity and 25(OH)D levels (r = −0.173, *p* = 0.249; all-age groups), although the mean level of this factor was highest in mild AD (25.7 ± 8.1).

Currently, there is no specific dosing regimen for vitamin D in AD. Authors describe doses of 5000 IU daily, 60,000 IU weekly, or varying doses ranging from 8000 to 16,000 IU weekly [9]. However, a meta-analysis by Park et al. [14] including 5 RCTs showed that vitamin D supplementation at a dose of >2000 IU/day can reduce the severity of AD, while supplementation at a dose of ≤2000 IU/day does not provide any benefit. Despite the growing number of studies, the lack of specific recommendations regarding dosage limits the ability to determine the role and effectiveness of vitamin D in the treatment of AD.

## 5. Observational Studies

The gradually increasing interest in the relationship between vitamin D and allergic/atopic diseases [1] has given rise to ongoing studies suggesting an association between circulating vitamin D levels and the severity of AD, as well as indicating that vitamin D exerts therapeutic effects in this condition [9]. This is of particular importance given the substantial burden that AD imposes on physical, psychological, and social health, creating an urgent need for the development of novel treatment strategies [112].

### 5.1. Promising Results

Research [103], which assessed AD severity in relation to vitamin D supplementation, demonstrated an inverse association. The two-year prospective trial enrolled 152 patients of both sexes, ranging in age from neonates to adults. Disease severity was quantified using SCORAD index. At baseline, 116 participants had insufficient serum 25-hydroxyvitamin D levels, and 49 of these were frankly deficient. Those with adequate vitamin D status exhibited significantly lower SCORAD scores (*p* = 0.04). Participants with suboptimal vitamin D received a six-month supplementation regimen. After three months, their mean serum 25-hydroxyvitamin D concentration rose to 35.9 ng/mL, and their SCORAD score fell from 19.4 pre-supplementation to 12.3 post-supplementation (*p* < 0.001). Despite this overall improvement, 20 children—predominantly preschool-aged and treated during the winter months—showed no change. Nevertheless, the study supports that vitamin D supplementation can effectively attenuate AD exacerbations by modulating cutaneous inflammatory mechanisms.

A study [104] was conducted that involved the observation of fifty pediatric patients with AD who used vitamin D as a dietary supplement. The participants were divided into two groups: the first group consisted of children with a high SCORAD index (above 40), while the second group had lower SCORAD scores. Children with severe AD received higher doses of vitamin D to alleviate symptoms. After 20 days of vitamin D treatment, the difference in SCORAD scores between the two groups was no longer statistically significant (*p* = 0.649), and this remained unchanged throughout the supplementation period (*p* = 0.474). These findings suggest that vitamin D may be effectively used as a therapeutic agent in the treatment of AD in children.

To avoid numerous discrepancies and limitations found in previous studies, Mohamed et al. [113] conducted a case–control study in a pediatric population, including one hundred children with AD and one hundred and one healthy children as a control group. Serum levels of calcidiol were measured in all participants to assess vitamin D deficiency and its association with the prevalence of AD. The serum 25(OH)D levels were significantly lower in patients with AD compared to healthy controls (mean 22.6 *vs*. 35.1 ng/mL, *p* < 0.001). The prevalence ratio (PR) with 95% confidence intervals (CI) for AD, when comparing children with moderate and low vitamin D levels to those with optimal levels, was 3.11 (1.91, 5.06) and 4.77 (2.99, 7.60), respectively. A 37% higher prevalence of AD was observed for every 10 ng/mL decrease in vitamin D levels. The unadjusted risk ratio for AD in vitamin D-deficient individuals versus non-deficient individuals was 2.26 (1.83, 2.78). Moreover, the PR for AD in male participants with moderate and deficient vitamin D levels compared to those with optimal levels was 3.38 (1.21, 9.40) and 5.20 (1.91, 14.13), respectively. Among females, the corresponding values were 1.32 (0.96, 1.83) and 1.49 (1.04, 2.14). The study concluded that vitamin D deficiency is indeed associated with both the presence and severity of AD and that these associations show a clear sex-specific pattern.

The onset of allergic sensitization in early childhood may potentially increase the likelihood of AD developing concurrently [114]. Given the established association between vitamin D levels and specific allergic sensitizations, a study [115] was conducted to identify the influence of vitamin D on allergen sensitization and AD at various stages of childhood. This prospective study included a total of 222 children at the ages of 0.5, 2, and 4 years. Of these, 94 were healthy and served as the control group, while 128 children were diagnosed with AD. The results showed that children with AD had significantly lower serum calcidiol levels at ages 2 and 4 compared to the control group (*p* < 0.001). Moreover, children with AD at the age of 0.5 years (*p* < 0.001) and at 4 years (*p* < 0.01) exhibited a higher prevalence of food allergies. When comparing children with serum calcidiol levels >30 ng/mL to those with levels <20 ng/mL, the latter group showed a relatively higher frequency of food allergies at 0.5 years and dust mite sensitization at 2 years of age. Thus, vitamin D deficiency is indeed associated with both AD and a higher prevalence of allergen sensitization in early childhood. This is likely due to the role of vitamin D in modulating the immune response to allergens, thereby contributing to the development of AD.

To determine whether maternal vitamin D levels are associated with the development of AD, numerous studies, including observational studies, have been conducted. One such study is trial [116], which involved 4051 pregnant women carrying singleton pregnancies. In the first trimester, 581 women were vitamin D deficient, 1790 had insufficient levels, and 1680 had sufficient levels of the vitamin. During pregnancy, some mothers reported using vitamin D supplements or multivitamins containing vitamin D. At six months postpartum, AD was diagnosed in 555 infants (13.7%). Among these infants with skin disease, the average maternal serum calcidiol concentration during the first trimester was significantly inadequate (<50 nmol/L, *p* = 0.0012). Compared to mothers with adequate 25(OH)D levels, those with deficiencies had a 53% higher risk of their child developing AD within six months (RR: 1.53, 95% CI: 1.24–1.88). Additionally, inverse probability of treatment weighting (IPTW) analysis revealed a 154% increased risk of AD at six months (RR: 1.54, 95% CI: 1.22–1.93). Use of multivitamins was associated with a reduced incidence of the condition (RR: 0.79, 95% CI: 0.67–1.98), and vitamin D supplementation alone was even more protective (RR: 0.51, 95% CI: 0.37–0.71) when compared to mothers who did not take any supplements. A similar protective effect against the development of AD was observed in mothers who were vitamin D deficient in the first trimester but took either multivitamins or vitamin D supplements alone (RR: 0.72, 95% CI: 0.53–0.97 and RR: 0.44, 95% CI: 0.26–0.74, respectively). These findings, along with other results, suggest that maternal calcidiol deficiency during the first trimester of pregnancy is associated with an increased risk of AD in infants. However, this potential risk can be mitigated through the use of vitamin D supplements or multivitamin supplementation during pregnancy.

Despite the findings of previous studies, there are observational studies that report opposite results. A prospective cohort study [117] assessing how maternal serum calcidiol concentration during pregnancy influences the development of AD in children showed that higher vitamin D levels may increase the risk of manifestation of this condition. The study included 456 pregnant women. Among all infants born, 121 (26.5%) developed AD before the age of one. The median maternal serum calcidiol concentrations were 16.1 ng/mL in the first trimester, 16.3 ng/mL in the second trimester, and 14.0 ng/mL in the third trimester. The association between higher calcidiol levels throughout pregnancy and increased occurrence of AD was most pronounced during the first trimester (per ln unit increase: adjusted OR = 1.93, 95% CI: 0.96, 3.88) and the second trimester (per ln unit increase: adjusted OR = 1.72, 95% CI: 0.93, 3.19). The weakest association was observed in the third trimester (per ln unit increase: adjusted OR = 1.48, 95% CI: 0.87, 2.53). Taken together, the findings of this study suggest that maternal vitamin D levels in humans exceeding normal reference ranges may be associated with an increased risk of early-onset AD in infants.

To evaluate the expression of various proteins involved in maintaining the skin barrier and vitamin D metabolism in AD, a cross-sectional, exploratory study [118] was conducted in which the effect of different vitamin D levels on the expression of these proteins was simultaneously examined. The expression of proteins related to skin barrier maintenance and vitamin D metabolism was evaluated in 22 adult participants with AD, 17 of whom had the disease at moderate to severe levels. Individuals with serum 25(OH)D levels below 30 ng/mL (16 participants) showed increased expression of CYP24A (*p* = 0.054), alpha-catenin (*p* = 0.043), and haptoglobin (*p* = 0.033) compared to those with 25(OH)D levels above 30 ng/mL (6 participants). Similarly, participants with calcidiol levels below 20 ng/mL (6 participants) exhibited significantly higher expression of haptoglobin (*p* = 0.021). Additionally, it was identified that when IgE levels were above 100 IU/mL, there was an increased expression of VDR (*p* = 0.007), as well as occludin-1 (*p* = 0.011) and beta-catenin (*p* = 0.041). Vitamin D, through its observed negative correlation with the expression of specific tight junction proteins, indicates a multidimensional relationship between the vitamin D metabolic process and the integrity of the skin barrier during the active phase of AD. Moreover, as the statistics show, vitamin D plays an essential role in regulating antimicrobial peptides such as cathelicidin (log-fold changes = 1.79; *p* = 0.05) and the VDR pathway, which contributes to alleviating chronic inflammation. 

An another study [105] focused on evaluating the relationship between vitamin D deficiency and the severity of eczema in children and young adults from Bangladesh living in London. In a cohort study based on data collected from various sources, 681 participants were involved. Approximately 285 of the 338 participants (49.6%) whose serum calcidiol levels were available had a deficiency or insufficient levels of the minimum 25(OH)D concentration. This phenomenon can be explained by the fact that South Asian individuals currently living in the United Kingdom exhibit lower vitamin D levels compared to white individuals also residing in the UK [119,120]. The median EASI score was 4.3 (1.5, 10.4), which was higher in individuals with deficiency and insufficient calcidiol levels compared to those with adequate levels of calcidiol. Both the lowest and most current 25(OH)D levels were inversely correlated with the EASI score (R^2^ = −0.24, *p* < 0.001; R^2^ = −0.11, *p* = 0.0035). A low median of the lowest and current calcidiol levels, along with an EASI > 10, was present in 178 patients. After adjusting for confounding factors, EASI > 10 was significantly associated with vitamin D deficiency (OR 3.26, 95%CI 1.35, 8.60; *p* = 0.012), as well as the use of topical steroids on the face, neck, and body, and the topical use of calcineurin inhibitors on the face and neck. The compiled and analyzed data showed a connection between low 25(OH)D levels and exacerbation of eczema in the Bangladeshi population in London.

Interestingly, the authors of the Croatian report [121] also described vitamin D deficiency in patients with AD, but found no significant differences depending on age, gender or living environment, nor a correlation with total IgE concentration. The study included 157 adult patients, including 51 with AD, and lasted three months. Patients received vitamin D3 supplementation in two doses: 1000 IU daily for mild deficiency or 1400 IU daily for severe deficiency. After the supplementation, vitamin levels increased significantly, and clinical improvement was noted, but the differences were not statistically significant (69.8 vs. 58.1, *p* = 0.428). Lugović-Mihić et al. indicated that vitamin supplementation leads to improvement in most patients without worsening their condition, which indicates the possibility of no side effects [121].

### 5.2. Genetic Polymorphism Assessment

Various single nucleotide polymorphisms (SNPs) in VDR and CYP24A1 are correlated with AD (Table 1) [122,123]. A genotyping study [124] was conducted on 246 participants, including 143 patients with AD and 103 healthy controls. The analysis included 10 different polymorphisms in the VDR gene (rs2239185, rs1544410, rs7975232, rs2238136, rs3782905, rs2239179, rs1540339, rs2107301, rs2239182, and rs731236) and 2 polymorphisms in CYP24A1 (rs2248359 and rs2296241). Significant correlations were observed for 2 VDR polymorphisms related to the disease. The first, rs2239182-C/C in the dominant model, reduced the risk of AD by 66% (OR 0.34, 95% CI 0.13–0.87; *p* = 0.03), and in the dominant model (presence of the C allele), it reduced the risk by 58% (OR 0.42, 95% CI 0.18–0.90; *p* = 0.03). The second, rs2238136, showed the opposite effect. The rs2238136 polymorphism is a risk factor for AD in co-dominant, dominant, and over-dominant models. This SNP increases the risk of the disease 2 or 3 times depending on the allele and model. The GCC haplotype (rs2239185-G, rs1540339-C, and rs2238136-C) also reduced the risk of AD by 49% (OR 0.511, 95% CI 0.232–0.939; *p* = 0.04). For the CYP24A1 gene, both SNPs (rs2296241-AA and rs2248359-TT) were associated with higher-than-normal HDL cholesterol levels. The study thus presents the existence of a bidirectional relationship between certain genetic factors related to vitamin D function and the development of AD. It also emphasizes the complexity of the genetic background affecting atopic skin disease. 

### 5.3. No Expected Research Results

Not all studies show a link between vitamin D and AD (Table 2). Shimizu et al., in their study “Japan Environment and Children’s Study” [129], investigated whether maternal vitamin D intake during pregnancy is associated with the symptoms of allergic diseases in children at the age of 3. The cohort study included data from 73,309 mother-child pairs. The frequency of various allergic symptoms in 3-year-old children was assessed, including current wheezing, current rhinitis, current nasal and conjunctival symptoms, current eczema, asthma, hay fever, and AD. The respective prevalences were 17.2%, 29.7%, 3.8%, 15.2%, 9.6%, 3.7%, and 11.0%. Risk ratios for current rhinitis were significantly lower in individuals with medium (approximately 3.9 µg/day), moderately high (approximately 5.5 µg/day), and high (approximately 10.5 µg/day) vitamin D intake compared to those with low (approximately 1.1 µg/day) vitamin D intake. However, regarding current wheezing, eczema, asthma, and AD, no clear trend was observed between the morbidity rates and the amounts of vitamin D consumed by the mother during pregnancy. In AD, a slight increase in the morbidity rate was noted in individuals with high vitamin D intake (OR = 1.08, 95% CI: 0.98–1.18, *p* = 0.109). Therefore, this study only demonstrated that maternal vitamin D intake during pregnancy is associated with a lower risk of nasal allergies in children. No significant connections were found between AD and various amounts of vitamin D intake.

## 6. Animal Research

Mouse models prove to be critical and indispensable for investigating and optimizing the efficacy of pharmacological agents in a wide array of diseases. A significant advantage of employing murine-induced models is the ability to precisely elucidate the mechanisms not only underlying symptom exacerbation but also the therapeutic amelioration of dermal lesions in AD (Table 3). Nonetheless, due to the anatomical disparities between murine and human skin, certain limitations in the translatability of these findings persist [131].

For instance, a study conducted in 2023 [98] verified the effect of vitamin D supplementation on AD induced by 2,4-dinitrochlorobenzene (DNCB) in female BALB/c mice. The study lasted for 18 days and utilized calcifediol, which is metabolized in vivo to calcitriol. Initially, clinical skin biopsy samples demonstrated that the epidermis exhibited reduced levels of VDBP and VDR proteins compared to normal skin (*p* < 0.001, n = 3), resulting in a vitamin D deficiency in the skin. Subsequently, the efficacy of calcifediol both alone and in combination with dexamethasone was identified in reducing inflammatory infiltration and edema in the ears of mice with AD. The thickness and weight of the mouse ears significantly decreased (*p* < 0.0001, *p* < 0.05, n = 5). Additionally, calcifediol and dexamethasone reduced erythema and scaling (*p* < 0.001, *p* < 0.01, n = 5) during AD on the dorsal region of the mice. In the experiment, calcifediol inhibited the phosphorylation of the STAT3/AKT1/mTOR signaling pathway (*p* < 0.0001, n = 6), improved the expression of the previously mentioned VDBP and VDR proteins (*p* < 0.05, n = 5), and attenuated the expression of pro-inflammatory factors in DNCB-induced atopic skin. It was also observed that the expression of AQP3, which is excessively expressed in AD and leads to transepidermal water loss, decreased in this experiment (*p* < 0.0001, n = 5). The AQP3 protein may cause skin dryness and disrupt the barrier function of the skin [134]. All the results of the study thus indicate the protective role of vitamin D in AD.

In a similar study [132] using female BALB/c mice, researchers investigated how vitamin D interacts with allergic responses. Mice exposed to sensitizing chicken egg albumin (OVA) for 70 days developed mild epidermal thickening and scaling similar to that observed in human AD. The expression of RXRα, enzymes involved in regulating VDR activity, and VDR target genes such as CYP24A1 were increased in mice exposed to OVA. The study demonstrates that, contrary to the common belief that vitamin D is reduced in AD, the opposite situation occurs in this case. Vitamin D is present in increased amounts at the site of inflammation, which may result in decreased serum levels. This suggests that the deficiency of vitamin D in the serum of patients with AD might stem from its intensive utilization at inflammation sites rather than from an overall deficiency in the body.

To assess the therapeutic efficacy of crisaborole in combination with vitamin D against allergic contact dermatitis (ACD), an experiment [133] was conducted. Crisaborole, as a non-steroidal topical phosphodiesterase 4 inhibitor, can be used in the treatment of AD by regulating inflammatory cytokines that are more active during inflammation [135]. The study used HaCaT cells and 40 male C57BL/6 mice, most of which were sensitized with DNCB to induce ACD. In HaCaT cells exposed to TNF-α/IFN-γ, the mRNA expression of IL-17A, IL-6, IL-4, TNF-α, CCL2, and CCR2 was reduced by treatment with vitamin D alone, crisaborole alone, and the combination of vitamin D and crisaborole. The greatest reduction in expression was observed when vitamin D and crisaborole were used together. A similar phenomenon was observed in the mice for the expression of caspase-1, IL-1β, IL-6, IL-4, TNF-α, iNOS, IL-17A, CCL2, and CCR2. Additionally, vitamin D and crisaborole decreased edema, transepidermal water loss, epidermal thickness, and mast cell infiltration at the sites of skin inflammation. In this study, vitamin D, as an adjunct to crisaborole, proved to be crucial and decisive in immunoregulation and in improving the condition of the skin after inflammatory damage

## 7. RCTs

Over the past decade, a growing number of RCTs have emerged to critically evaluate the role of vitamin D supplementation as a preventive and therapeutic strategy for AD. While earlier observational studies provided compelling associative data linking vitamin D deficiency to AD prevalence and severity, it is only through rigorously conducted interventional studies that the field has begun to untangle the causality, dose responsiveness, and clinical relevance of vitamin D repletion. These studies, often focused on pediatric populations where disease burden is high and systemic intervention is feasible, provide valuable information on timing, immune phenotype, host genotype, and vitamin D pharmacodynamics. In Table 4, we provide an overview of these key studies, with particular emphasis on the study population, interventions, and outcomes.

### 7.1. Prenatal Vitamin D Supplementation and the Risk of AD in Children

The MAVIDOS trial [4] published in 2022 describes a prenatal vitamin D intervention. This multicentre, double-blind, placebo-controlled trial enrolled 703 pregnant women with intermediate-range serum 25(OH)D concentrations (25–100 nmol/L). Participants were randomized in a 1:1 ratio to receive either 1000 IU/day of oral cholecalciferol (n = 352) or placebo (n = 351), beginning at 14 weeks gestation and continuing until delivery. Follow-up assessments were performed at 12, 24, and 48 months postpartum to determine the incidence of clinically diagnosed AD in offspring.

At 12 months, children of mothers who received vitamin D exhibited a significantly lower risk of developing AD (OR: 0.57; 95% CI: 0.33–0.98; *p* = 0.04), suggesting a temporally confined but clinically relevant immunoprotective effect of prenatal vitamin D exposure. This effect was not sustained at later timepoints, with the odds ratios approaching null and *p*-values losing statistical significance by the 24- and 48-month evaluations. However, subgroup analysis revealed that infants who were breastfed for more than one month experienced a more pronounced benefit (OR: 0.48; *p* = 0.03), implying a synergistic interaction between maternal-fetal vitamin D transfer and immunomodulatory constituents of breast milk, such as transforming growth factor-β and secretory IgA. Notably, genotyping of maternal vitamin D metabolism genes (CYP2R1, DHCR7, and CYP24A1) showed no significant associations with outcome, discounting common SNP-based variation as a major driver and instead implicating epigenetic regulation or microbiome-mediated mechanisms as more plausible mediators of immune imprinting.

These findings have profound translational implications. They suggest that timing of exposure—specifically in utero and early neonatal periods—may critically shape Th2/Th17 trajectory and barrier tolerance, as well as that the maternal supplementation might be an accessible public health measure to transiently reduce AD burden in the first year of life. However, the waning effect over timepoints to the need for postnatal maintenance strategies and perhaps stratification of benefit by breast milk composition, delivery mode, or environmental microbial exposures.

### 7.2. Vitamin D Supplementation as Adjunctive Therapy in Severe Pediatric AD

In pediatric patients with established AD, interventional trials have shown more robust and consistent clinical efficacy, particularly in those with moderate to severe cases of the disease. Mansour et al. [3] performed a randomized, double-blind, placebo-controlled trial in 86 children aged 5 to 16 years diagnosed with severe AD, defined by clinical EASI scores and resistance to emollient therapy. Participants were randomized to receive either 1600 IU/day of oral cholecalciferol (n = 44) or placebo (n = 42), in combination with standardized 1% topical hydrocortisone, for a total duration of 12 weeks.

The results demonstrated a statistically and clinically significant improvement in the vitamin D group. The mean percentage reduction in EASI score was 56.4% compared to 42.1% in the placebo group (*p* = 0.039). Furthermore, 38.6% of children in the intervention arm achieved EASI-75 (a ≥ 75% reduction in baseline EASI), versus only 7.1% in the control group. Importantly, a strong correlation was observed between serum 25(OH)D increase and clinical improvement (r = 0.6; *p* = 0.005), providing evidence for a dose-dependent therapeutic effect. These findings support the notion that vitamin D’s clinical benefit in AD likely extends beyond classical calcium homeostasis, involving regulation of innate antimicrobial peptides (e.g., cathelicidin LL-37), restoration of filaggrin expression, as well as modulation of IL-4/IL-13-driven inflammation. From a therapeutic standpoint, these data suggest that correcting subclinical vitamin D deficiency may augment standard corticosteroid efficacy and facilitate more rapid disease control in children with active inflammation.

### 7.3. High-Dose Vitamin D Supplementation and Immune Biomarkers

To address the dose–response and biomarker correlation of vitamin D therapy in AD, Borzutzky et al. [6] designed a mechanistically oriented RCT involving 101 children with active AD (mean age 6.3 ± 4.0 yrs). Participants were randomized to receive weekly age-adjusted high-dose vitamin D3 (8000–16,000 IU/week; n = 53) or placebo (n = 48) for six weeks. In addition to clinical assessments (SCORAD), the trial evaluated serum levels of eosinophils, total IgE, specific IgE, and key Th2 chemokines (CCL17, CCL22, and CCL27), along with genotyping for vitamin D receptor polymorphisms.

Although the intervention group achieved a statistically significant increase in serum 25(OH)D levels (+43.4 ± 34.5 nmol/l vs. +2.3 ± 21.2 in placebo; *p* < 0.001), no difference in SCORAD reduction was detected between groups (−5.3 ± 11.6 vs. −5.5 ± 9.9; *p* = 0.91). Likewise, no changes were observed in eosinophils counts, IgE concentrations, or Th2 chemokine levels. Analysis of VDR SNPs (FokI, ApaI, TaqI) revealed no effect modification. However, post hoc stratification identified a subset of participants with intra-individual increases in 25(OH)D > 20 ng/mL who experienced greater symptomatic improvement, suggesting the presence of a vitamin D-responsive endotype within the broader AD population. These findings argue for precision-based dosing strategies and call into question the utility of fixed-dose regimens in heterogeneous cohorts.

### 7.4. Vitamin D and Synbiotics in Early Infancy

The potential convergence between vitamin D metabolism and gut microbiota in AD pathophysiology was examined by Aldaghi et al. [5], who conducted a triple-arm RCT in 81 infants under 12 months of age. Participants were randomized into three groups: 1000 IU/day of vitamin D3 (n = 27), a multistrain synbiotic formulation (n = 27), or standard care (n = 27), administered over eight weeks. SCORAD scores were measured at baseline and follow-up.

Both vitamin D and synbiotic groups demonstrated statistically significant reductions in SCORAD compared to controls (vitamin D: *p* = 0.001; synbiotics: *p* < 0.001), with no difference in efficacy between the active treatment arms. These results support the hypothesis that both interventions modulate overlapping immunological circuits—most notably the gut-skin axis, via short-chain fatty acid signaling, regulatory T cell induction, and attenuation of epithelial permeability. The trial adds to the emerging narrative that early-life microbial and metabolic programming may offer a critical window for durable immunologic intervention in AD.

## 8. Therapeutic Comparison: Vitamin D and Other Modalities

The underlie of AD treatment consists of anti-inflammatory drugs. Such agents include primarily topical corticosteroids, topical calcineurin inhibitors, topical phosphodiesterase-4 inhibitors, and oral Janus kinase (JAK) inhibitors [136]. Standard therapy does not always produce the expected effects or may be associated with adverse effects, which necessitates the use of more advanced treatment methods. In moderate and severe AD, where resistance to first-line topical treatment occurs, biological and small-molecule drugs have been developed that act directly on pathogenic AD pathways [137,138]. Examples of such drugs include dupilumab, tralokinumab, lebrikizumab, and JAK inhibitors. Cyclosporine and phototherapy based on ultraviolet radiation are also used to alleviate disease symptoms [138,139]. These types of therapies are characterized by an acceptable safety profile and clinical efficacy [137,138,139].

Despite minor side effects and generally safe use in AD, vitamin D is proposed as an adjunct therapy for patients with moderate or severe forms of this disease. This is due to its lower efficacy compared to compounds such as upadacitinib or abrocitinib, which demonstrate the greatest impact in patients with AD [140,141]. Vitamin D is characterized by symptom-relieving properties comparable to the action of probiotics [142]. Similarly, vitamins D and E possess therapeutic properties for AD, simultaneously exhibiting a synergistic effect when used together [143]. Its effects in combination therapy with other drugs are currently under investigation.

Animal models are crucial for studying disease mechanisms and evaluating potential therapeutic agents [144]. In the study by Alosaimi et al. [100], the efficacy of AD treating with a cream containing vitamin D (0.0003%) versus a cream containing betamethasone (0.1%) was compared in 35 male BALB/c mice. AD was induced by ovalbumin. Mice treated with vitamin D or betamethasone following ovalbumin exposure showed a significant decrease in levels of IgE, IL-5, and filaggrin, as well as a significant increase in IL-4 and IL-13 compared to the group given ovalbumin alone (*p* < 0.05). Moreover, betamethasone exhibited slightly better efficacy in decreasing or increasing the analyzed parameters compared to the effects of vitamin D. In addition, the improvement in skin appearance was greater in mice treated with betamethasone than in those treated with vitamin D. In another study [133], the therapeutic effect of vitamin D and crisaborole—both applied separately and in combination—on skin inflammation was examined. Using 40 C57BL/6 mice, it was shown that expression of proteins such as caspase-1, IL-1β, IL-6, IL-4, TNF-α, iNOS, IL-17A, CCL2, and CCR2 was reduced under the influence of the tested substances (*p* < 0.01), with the weakest effect observed for vitamin D alone, a stronger effect for crisaborole, and the most pronounced reduction in expression occurring in the combined therapy. Both experiments indicate that vitamin D possesses anti-inflammatory properties similar—but somewhat inferior—to the corticosteroid betamethasone. Additionally, vitamin D in combination with crisaborole may exert a synergistic effect in the treatment of AD.

Among RCTs, there are also comparisons of the efficacy of therapies with vitamin D. The authors of a study published in 2020 [3] assessed whether vitamin D supplementation supports basic therapy with topical hydrocortisone cream (1%) for 12 weeks. In the group of 42 children receiving only the baseline therapy, the mean EASI score was 27.47, whereas in the group of 44 children receiving vitamin D, the mean EASI score was 20.42 (*p* = 0.035). In another experiment [5], the effect of a multistrain synbiotic and vitamin D3 supplements—both applied separately and in combination—on skin inflammation was examined. In the control group, patients underwent routine treatment using corticosteroids, emollients (Eucerin), and antihistamines. The second group of participants, in addition to routine treatment, received synbiotics. In the third group, patients had routine treatment plus vitamin D3 supplementation. After two months, the mean SCORAD score significantly decreased both in the synbiotic group (−13.90; *p* < 0.001) and the vitamin D3 group (−12.38; *p* = 0.001) compared to the control group. The obtained data indicate that vitamin D plays an important supportive role in improving the basic treatment of AD.

## 9. Discussion

Although scientific interest in vitamin D as a potential modulator of immune-related disorders, including AD, has increased markedly in recent years, the overall body of evidence remains inconclusive. These discrepancies can be attributed not only to variations in study methodologies but also to the influence of individual patient factors—such as baseline serum 25-hydroxyvitamin D [25(OH)D] levels, distinct immunological profiles, and underlying genetic and environmental determinants.

Several observational studies have consistently reported that lower circulating 25(OH)D levels are associated with heightened AD severity, as measured by validated clinical indices including SCORAD and EASI [26,27,28,108]. Furthermore, vitamin D supplementation—particularly in children and in patients with moderate to severe disease—has been linked to clinical improvement. These outcomes have been corroborated by findings from meta-analyses of randomized controlled trials [9,10].

In the study conducted by Mansour et al. [3], children with severe AD, who received standard treatment with topical corticosteroids combined with a daily dose of 1600 IU of cholecalciferol for 12 weeks, demonstrated significant clinical improvement. A greater reduction in EASI scores was observed in the intervention group (56.44% *vs.* 42.09%; *p* = 0.039), along with a higher rate of EASI-75 response. A statistically significant positive correlation was found between the increase in serum 25(OH)D levels and the degree of clinical improvement (r = 0.6; *p* = 0.005), suggesting that therapeutic efficacy may depend on baseline vitamin D status.

Parallel findings in animal models, including DNCB-induced AD in BALB/c mice, showed that calcifediol inhibited activation of inflammatory signaling pathways (STAT3/AKT/mTOR), reduced the expression of AQP3—a protein associated with transepidermal water loss—and upregulated the expression of VDR and VDBP [98,134]. These results indicate that vitamin D exerts not only systemic but also localized effects within the skin, which may be particularly relevant in the management of chronic inflammatory dermatoses.

On the other hand, some cross-sectional and prospective studies have failed to demonstrate a statistically significant association between serum 25(OH)D levels and AD severity [109,110,111,124]. Such inconsistencies underscore the need for cautious interpretation of existing data, as a wide range of confounding factors—including seasonal variation, UVB exposure, nutritional status, age, comorbidities, medication use, and socioeconomic conditions—may influence outcomes.

Furthermore, existing evidence suggests that prenatal vitamin D supplementation may exert a limited and time-dependent effect on the risk of AD in offspring. The MAVIDOS trial [4] showed a 43% reduction in the incidence of AD at 12 months among children whose mothers received cholecalciferol during pregnancy (OR: 0.57; *p* = 0.04), although this protective effect did not persist at 24 and 48 months. Interestingly, the observed benefit was more pronounced in infants who were breastfed for more than one month, suggesting a possible synergistic role of immunomodulatory components in human milk.

Genetic studies further highlight the role of specific single nucleotide polymorphisms (SNPs) in the VDR and CYP24A1 genes as potential biomarkers of AD susceptibility [124]. The C allele of rs2239182 was associated with a 66% lower risk of developing AD in a dominant model, whereas the rs2238136 variant was linked to more than a twofold increase in risk. These genetic differences may help explain the variability in clinical trial outcomes and point to the relevance of personalized supplementation strategies.

Additionally, recent findings indicate differential expression of proteins involved in vitamin D metabolism and epidermal barrier function—such as occludin-1, β-catenin, and haptoglobin—depending on 25(OH)D levels and total IgE concentrations [118]. This suggests that vitamin D may influence the inflammatory skin microenvironment through multiple mechanisms, beyond its well-established immunoregulatory properties. Against this backdrop, the concept of a “vitamin D–responsive endotype” of AD has emerged—referring to a subset of patients in whom clinical response to supplementation is modulated by immunological phenotype, genetic background, degree of deficiency, and local VDR activity. This approach may facilitate the development of more individualized therapeutic strategies based on molecular diagnostics, immune profiling, and assessment of cutaneous and gut microbiota.

## 10. Conclusions

The collective data presented in this review suggests that vitamin D may serve as a valuable adjunctive therapy in the management of AD, particularly in patients with documented deficiency and moderate to severe disease. Both clinical and experimental data support the immunomodulatory and barrier-restorative properties of vitamin D, including its role in down-regulating Th2 and Th17 pathways, enhancing epidermal protein expression, and modulating local inflammatory responses within the skin microenvironment.

RCTs have demonstrated beneficial outcomes following vitamin D supplementation, especially in pediatric populations, while prenatal interventions may temporarily reduce the risk of early-onset AD in offspring. However, findings remain partially inconsistent due to heterogeneity in study designs, population characteristics, intervention protocols, and the influence of environmental and genetic modifiers.

The emerging identification of vitamin D-responsive endotypes highlights the need for a more personalized approach to therapy, taking into account baseline 25(OH)D levels, immunological phenotypes, and relevant single-nucleotide polymorphisms (e.g., VDR, CYP24A1). The integration of molecular diagnostics, immune profiling, and microbiome assessment may help stratify patients and optimize treatment efficacy.

Further high-quality, stratified clinical trials are warranted to validate these findings, define optimal dosing strategies, and explore the long-term impact of vitamin D supplementation on disease trajectory, flare frequency, and skin microbiota composition in AD.

## Figures and Tables

**Table 1:** The influence of various vitamin D receptor gene polymorphisms on the risk and severity of AD identified since 2020.

VDR Polymorphisms	Observations	Conclusion	Reference
rs2238136	Individuals carrying the rs2238136-C/T genotype have a 2.94- to 3.09-fold increased risk of developing AD (*p* = 0.2). Under the dominant model, the presence of a single T allele elevates the risk of AD by 2.7 times (*p* = 0.2)	↑ risk or severity of AD	[124,125,126,127,128]
rs2228570	The FoKI polymorphism showed a significant association with an increased risk of AD across multiple genetic models, including the co-dominant model (*p* = 0.000), the recessive model (*p* = 0.000), the dominant model (*p* = 0.028), and the allele model		
rs4516035 (homozygote)	The presence of two or more homozygous VDR polymorphisms was linked with a higher risk of developing allergic reactions (*p* = 0.0003)		
rs731263	A significant association was observed between the VDR polymorphism and increased blood eosinophil levels in patients with AD (*p* < 0.05)		
rs11168293 (genotype GG)	Atopic patients with the GG variant of the VDR gene exhibited significantly higher IgE levels compared to those with the GT variant (*p* = 0.022). Additionally, association was identified between the GG genotype of this VDR polymorphism and elevated blood eosinophil levels in individuals with AD (*p* = 0.039)		
rs7041 (genotype CC)	The CC genotype was linked to increased IL-35 levels and Th17 and Th2 cells compared to the other genotype (*p* < 0.05).		
rs3733359 (genotype GG)	Individuals carrying the GG genotype showed significantly lower IL-10 levels compared to those with the GT genotype (*p* < 0.05)		
rs2239182	The rs2239182-C/C genotype was bond with a protective effect, lowering the risk of AD by 66% (*p* = 0.3).	↓ risk or severity of AD	[124,126,128]
rs2239185-G	Seemed to confer a protective effect, decreasing the risk of AD by 49% (*p* = 0.4)		
rs1540339-C	Seemed to confer a protective effect, decreasing the risk of AD by 49% (*p* = 0.4)		
rs2238136-C	Seemed to confer a protective effect, decreasing the risk of AD by 49% (*p* = 0.4)		
rs4516035 (heterozygote)	A lower odds ratio for AD onset was observed in individuals with heterozygote for this VDR polymorphism (*p* = 0.012)		
rs11168293 (genotype TT)	People carrying the TT genotype showed elevated IL-10 levels compared to individuals with other rs11168293 genotypes (*p* < 0.05)		
rs4588 (genotype GG)	The GG genotype was linked with lower Th2 and Th17 cell levels compared to GT and TT genotypes in patients (*p* < 0.05)		

AD—atopic dermatitis; VDR—Vitamin D Receptor; ↑—increased; ↓—decreased.

**Table 2:** No link between vitamin D and AD—studies published since 2020.

Reference	Participants (n)	Participants with Vitamin D Deficiency (n)	Participants with Normal Vitamin D Level (n)	Characteristics of the Participants	Outcomes
[109]	41	31	10	25 men and 16 women of all ages with clinical diagnosis of AD	No significant association between serum level of vitamin D and the severity of AD
[129]	73,309	13,993 *	14,528 ** 14,743 #	Pregnant women	No noticeable association between maternal vitamin D intake and symptoms of AD in their 3-year-old children
[130]	223	223	0	Newborns at high risk of allergies	No statistically significant association between neonatal calcidiol levels and the development of AD

* Individuals with low vitamin D intake (1.1 µg/day); ** Individuals with moderate vitamin D intake (3.9 µg/day); # Individuals with high vitamin D intake (10.5 µg/day); AD—atopic dermatitis.

**Table 3:** Preclinical studies investigating the link between vitamin D and AD—studies published since 2020.

Reference	Mouse Model	Duration of the Supplementation	Vitamin D Dose	Observation Period	Outcomes
[98]	DNCB BALB/c 25 mice (divided into 5 groups)	From day 6 to day 18 *	5 µg/kg of calcifediol	From day 6 to day 18	Significant reduction in AD symptoms—the mechanism may be related to the reduction in aquaporin 3 (AQP3) expression, modulation of inflammation and chemokines, inhibition of activation of related inflammatory pathways and cell proliferation pathways, and thus repair of skin barrier function
[132]	OVA BALB/c 18 mice (divided into 3 groups)	-	-	70 days	Increased synthesis of 1,25VD3 in the skin of mice sensitized to systemic allergens, as well as systemic and local allergens Increased expression of the VDR 24-hydroxylase target gene in the skin of animals sensitized to both systemic (65-fold) and systemic and local allergens (726-fold)
[133]	DNCB C57BL/6 40 mice (divided into 5 groups)	From day 6 to day 14 of the study **	0.025 mg/kg	Every day from day 1 to 14	Partial alleviation of AD symptoms by vitamin D; significant increase in therapeutic efficacy when combined with crisaborole

* Once every two days (7 doses); ** Once a day; DNCB—2,4-dinitrochlorobenzene; OVA—ovalbumin; AD—atopic dermatitis.

**Table 4:** Characteristics of a randomized controlled trials published since 2020.

Reference	Clinical Trial Number	Population & Intervention	Key Findings & Statistical Outcomes
Mansour et al. (2020) [3]	NCT04468711	86 children (5–16 yrs) 1600 IU/day cholecalciferol + 1% hydrocortisone for 12 weeks	Mean EASI reduction: 56.4% (vit D) vs. 42.1% (placebo), *p* = 0.039. EASI-75 achieved 38.6% (vit D) vs. 7.1% (placebo), ρ = 0.6 between 25(OH)D increase and EASI improvement
El-Heis et al. (2022) [4]	ISRCTN 82927713 EudraCT 2007-001716-23	703 pregnant women 1000 IU/day cholecalciferol from gestational week 14 to delivery	Reduced AD risk at 12 months (OR = 0.57, 95% CI: 0.33–0.98, *p* = 0.04); no difference at 24/48 months. Protective effect stronger in infants breastfed >1 month (OR = 0.48, *p* = 0.03) No gene–vitamin D interactions
Aldaghi et al. (2022) [5]	IRCT20180626040248N1	81 infants <1 yr 1000 IU/day vitamin D3 or synbiotics for 8 weeks	Both synbiotics and vitamin D groups had significantly lower SCORAD vs. control (*p* = 0.001 and *p* < 0.001 respectively) No difference between treatment arms
Borzutzky et al. (2024) [6]	NCT01996423	101 children (mean age 6.3 ± 4.0 yrs) vitamin D3 for 6 weeks (age-adjusted: 8000–16,000 IU/week)	25(OH)D increased by +43.4 ± 34.5 nmol/L (vit D) vs. +2.3 ± 21.2 (placebo), *p* < 0.001. No difference in SCORAD reduction: −5.3 ± 11.6 (vit D) vs. −5.5 ± 9.9 (placebo), *p* = 0.91. No change in IgE, eosinophils, chemokines (CCL17, CCL22, CCL27). VDR SNPs (FokI, ApaI, TaqI) had no modifying effect

AD—atopic dermatitis; SCORAD—scoring atopic dermatitis index; EASI—eczema area and severity index; VDR—Vitamin D Receptor; SNPs—single nucleotide polymorphisms; CCL17, CCL22, and CCL27—key Th2 chemokines.

## Data Availability

All data is contained within the article.

## Abbreviations

AD—atopic dermatitis; SCORAD—scoring atopic dermatitis index; EASI—eczema area and severity index; TSLP—thymic stromal lymphopoietin; ILC2—type 2 innate lymphoid cells; VDBP—vitamin D-binding protein; VDR—vitamin D receptor; CYP24A1— 25-Hydroxyvitamin D-24-hydroxylase; SNP—single nucleotide polymorphism; DNCB—2,4-dinitrochlorobenzene; UVB—ultraviolet B radiation.
